# Evaluation of the Psychometric Properties of Jebsen Taylor Hand Function Test (JTHFT) in Italian Individuals With Multiple Sclerosis

**DOI:** 10.3389/fneur.2022.847807

**Published:** 2022-03-18

**Authors:** Anna Berardi, Giovanni Galeoto, Federica Pasquali, Viola Baione, Sebastiano Giuseppe Crisafulli, Marco Tofani, Matteo Tartaglia, Giovanni Fabbrini, Antonella Conte

**Affiliations:** ^1^Department of Human Neurosciences, Sapienza University of Rome, Rome, Italy; ^2^IRCSS Neuromed, Pozzilli, Italy; ^3^Sapienza University of Rome, Rome, Italy; ^4^Department of Public Health and Infectious Disease, Sapienza University of Rome, Rome, Italy

**Keywords:** disability, multiple sclerosis, occupational therapy, psychometric properties, rehabilitation

## Abstract

**Introduction:**

The Jebsen Taylor Hand Function Test (JTHFT) is a non-diagnostic assessment scale for hand and upper limb dexterity that is commonly used in various countries around the world for diseases, such as muscular dystrophy, stroke, spinal cord injury, Parkinson, carpal tunnel syndrome, and rheumatoid arthritis. This study aimed to evaluate the psychometric properties of the JTHFT in Italian adults with Multiple Sclerosis (MS).

**Materials and Methods:**

The test's internal consistency was evaluated with Cronbach's alpha, whereas its concurrent validity was evaluated by comparing the JTHFT with the Health Assessment Questionnaire (HAQ) and by calculating Pearson's correlation coefficient.

**Results:**

The JTHFT was administered to 29 Italians with MS. The Cronbach's alpha showed that the nondominant hand has a value of 0.76 and 0.91 for the dominant hand. Pearson's correlation coefficient showed significant correlations between JTHFT and HAQ.

**Discussion:**

The JTHFT is a reliable tool to evaluate the functionality of the upper limb and hand in patients with MS. This tool is useful for testing the effectiveness of a treatment in various diseases. The results obtained in this study are coherent with previous studies that are conducted in populations with different diseases. In particular, the correlation between JTHFT and HAQ showed that a disability related to the upper limbs can often have repercussions, not only on activities of daily living, but also on walking. Based on this correlation, the motor deficits that emerged may be linked to a brain marrow disease rather than a spinal disease, even if an essential deepening can confirm this hypothesis.

## Introduction

Multiple sclerosis (MS) is a chronic inflammatory demyelinating disease that affects the central nervous system. It is the main cause of progressive neurological disability (1). Individuals with MS usually present with neurological deficits, cerebellar symptoms, fatigue, and cognitive deficits. Previous studies showed that sensory function (85%), fatigue (81%), impaired hand function (60%), and mobility (50%) were the most frequently reported symptoms in the first year of the disease. A combination of symptoms results in disability, which often impairs the ability to perform activities of daily living (ADL) and social activities, resulting in reduced quality of life (2).

Nowadays, several studies highlight the importance of addressing the rehabilitation of upper limb function in people with MS. Recent funding has shown a high percentage of individuals reporting upper limb dysfunction, even in the early stages of the disease (3–6). Kierkegaard et al. found in 2012 (7) that manual dexterity (Nine Hole Peg Test) is an important predictor of overall activity and participation within the community (Frenchay Activity Index). The upper limb dysfunction in MS contributes to a decrease in the ability to perform ADL, resulting in reduced independence and quality of life (8). Upper limb dysfunction is, in addition to walking, fatigue, and cognitive deficits, one of the critical dysfunctions present in MS (2, 9).

During the pathology progression, the fatigue and sensory and motor disorders to the lower and upper limbs can reduce the independence in the ADL with consequent reduction of quality of life. Therefore, it is important to base a proper rehabilitation program after a careful evaluation based on interviews and observation of occupational performance, especially on the administration of scales that give an objective judgment to the patient's upper limb and hand clinical conditions. A systematic review reports the following assessment scales used for the upper limb in multiple sclerosis: Fugl Meyer Assessment, Motricity Index, Modified Ashworth Scale, Fahn tremor Rating Scale, the Nine-hole peg test (NHPT), the Box and Block test (BBT), Action Research Arm Test (ARAT), Jebsen Taylor Hand Function Test (JTHFT), Purdue Pegboard Test, ABILHAND, Motor Activity Log (MAL), Disabilities of the Arm, Shoulder, and Hand (DASH), and Manual Ability Measure-36 (MAM-36) (2).

The evaluation of upper limb and hand functions is essential to develop a proper rehabilitation program, to identify limits and residual abilities, and to monitor the progression of symptoms. Clinicians need clear and complete information to use when making patient care decisions; there is a need for standardization in the outcome assessment. These would benefit patients, researchers, and clinicians. Universally validated outcome measures are needed to allow comparisons across the practice (10).

The JTHFT is a non-diagnostic assessment scale for hand and upper limb dexterity that is commonly used in various countries around the world (11–20) for diseases, such as muscular dystrophy (21), Stroke, (15, 18) Spinal Cord Injury (22), Parkinson (23), carpal tunnel syndrome (24), and rheumatoid arthritis (12). The tool is already used for people with MS (25), however, its psychometric properties for this population have never been studied.

This scale is classified by the International Classification of Function (ICF) as an activity assessment tool, and it evaluates changes in functional activities (26). This feature is an advantage and the only scale that evaluates the dexterities of the hand and the upper limb solely concerningtime-based and most common daily activities, such as writing and power simulation.

In addition, the “participation” assessment tools assess the difficulties that a person may encounter in engaging in the activities of life. The Katz Basic and Instrumental ADL Index, the Frenchay Activities Index, and the Functional Independence Measure are frequently used outcome measures to assess the restriction at the “activity” and “participation” levels. These assessment tools, however, strongly influence the individual's ability to walk, perform transfers, and cognitive condition. In contrast, the JTHFT appreciates the ability to perform various ADL-like tasks that require the handling or transport of standardized small and large objects using different gripping functions (8).

Although developed by Jebsen et al. in 1969, it is still widely used in rehabilitation especially in assessing dexterity in daily activities (27). It is validated and used in many languages and many countries worldwide, and it also allows to compare the outcomes of different clinical studies. It is useful for evaluating the effectiveness of a treatment in various diseases and using the same outcome measure can define which intervention is more effective than others.

This study aimed to evaluate the Italian version of JTHFT (JTHFCT-IT)'s psychometric properties on a population of adults with MS.

## Materials and Methods

This study was carried out by a research group from the Sapienza University of Rome and the Rehabilitation & Outcome Measure Assessment (R.O.M.A.) Association (28–34).

### Participants

According to previous validation studies of the same assessment tool, the minimum number of participants considered was 25 (34). The sample was recruited from February to October 2020 in the Department of Human Neurosciences of Polyclinic Umberto I of Rome. Those at the neurology clinic with a diagnosis of MS (according to the McDonald's standard) (35) were invited to participate in the study. They had to have the ability to understand instructions and perform the scale's activities, to have a range of Expanded Disability Status Scale (EDSS) between 0 and 9, and shall not have comorbidities that affect upper limb's functionality.

All participants were informed about the procedures and purposes of the study, and those who were interested in participating in the study gave their written consent before inclusion (36, 37).

### Validation Procedures

Three clinicians (a neurologist and two occupational therapists) screened all patients of neurology's clinic and applied inclusion/exclusion criteria for recruitment. After being included, the raters recorded demographic and clinical characteristics and administered the tests. The tests were administered in the same order: JTHFT-IT, Jamar dynamometer (12), and the Health Assessment Questionnaire (HAQ).

### Tools

The JTHFT consists of seven elements administered with verbal instruction and standard verbal instruction modalities. The tasks are as follows: (1) write a 24-character sentence; (2) flip three 7.62 cm × 12.7 cm (3” x 5”) sheets, simulating turning a page; (3) collect small common items, including a penny, paper clips, and bottle caps, and place them in a container; (4) stackable pawns; (5) pick up small items with a spoon; (6) moving light cans; and (7) moving heavy cans. Tests are evaluated by recording the number of seconds it takes to complete each task. The increase in the time to complete the test correlates with the decrease in hand function. Each activity is initially done with the non-dominant hand, and then with the dominant hand.

The HAQ, developed in 1980, is among the first Patient-Reported Outcome Measures. The HAQ includes items that assess the upper extremity's fine movements, lower limb activities, and tasks that involve both the upper and lower limbs. It is composed of 20 items divided into eight categories, which represent a comprehensive set of ADLs: dressing, rising, eating, walking, hygiene, reach, grip, and usual activities. Each item is scored from 0 to 3, with higher scores indicating more disability (0 = without any difficulty; 1 = with some difficulty; 2 = with much difficulty; and 3 = unable to do). Scores of 0 to 1 generally represent mild to moderate difficulty, 1–2 represent moderate to severe disability, and 2 to 3 indicate severe to very severe disability (38).

### Data Analysis

The psychometric properties were assessed by following the Consensus-Based Standards for the Selection of Health Status Measurement Instruments (COSMIN) checklist (39).

The internal consistency was evaluated using Cronbach's alpha (α) to assess the items' correlation and the homogeneity of the scale; the coefficient must be at least 0.7 to indicate the acceptable homogeneity of all the items within a scale (13). The concurrent validity of the JTHFT-IT was evaluated by calculating the Pearson's correlation coefficient (ρ) between the test and dynamometer (40), and HAQ. The following values were considered in interpreting the results: ρ > 0.70 = strong correlation, 0.50 < ρ <0.70 = moderate correlation, and ρ <0.50 = weak correlation. Any *p* ≤ 0.05 were considered statistically significant. All statistical analyses were performed using the Statistical Package for the Social Sciences (SPSS) version 20.0 for Windows (13).

## Results

The scale was administered to 29 people, of whom 51.7% were women, with an average age of 47.59 years (9.57 in standard deviation). The demographic characteristics of the population are shown in Table 1.

**Table 1:** Demographic characteristics of the population.

	**Mean (Standard Deviation)**
Age	47.59 (9.57)
Years from diagnosis	13.82 (8.88)
**Gender**	**Frequency (%)**
Male	14 (48.3)
Female	15 (51.7)
**Occupation**	**Frequency (%)**
Unemployed	7 (24.1)
Employee	14 (48.3)
Freelancer	6 (20.7
Retired	1 (3.4)
Student	1 (3.4)
**EDSS**	**Frequency (%)**
0–2	4 (13.8)
2.5	7 (24.1)
3.0–4.5	3 (10.3)
5.5	2 (6.9)
6.0	4(13.8)
6.5	5 (17.2)
7.0–8.0	2 (6.9)

The Cronbach alpha value from the statistics showed an internal consistency of 0.76 for the non-dominant hand and 0.91 for the dominant hand. Table 2 shows the mean, SD, and Cronbach alpha values if one of the items of the scale is removed, higher results have been found for the dominant hand. The criterion validity and analysis of the JTHFT-IT results were performed in the dominant and non-dominant hand, where statistically significant variations with the HAQ were found in Tables 3, 4. These tables report the statistically significant correlations within all subscales and strong correlations (ρ > 0.70) for “pick up small objects”, “moving light cans,” and “moving weight cans”. The subscale of “grip” correlates only in the results of the dominant hand.

**Table 2:** Mean SD and Cronbach alpha values of one of the items of the scale.

		**Mean**	**Std. deviation**	**Cronbach's Alpha if item deleted**
Non dominant hand	Writing	52.15	30.23	0.79
	Turning pages	8.99	6.89	0.71
	Pick up small objects	11.12	5.51	0.73
	Simulate feeding	20.85	21.27	0.65
	Stacking Checkers	9.73	6.05	0.74
	Moving light cans	6.92	3.02	0.76
	Moving weight cans	7.19	3.74	0.76
Total alpha non dominant hand = 0.76				
Dominant hand	Writing	16.73	7.94	0.91
	Turning pages	6.93	4.04	0.91
	Pick up small objects	9.08	3.54	0.89
	Simulate feeding	13.54	8.08	0.89
	Stacking Checkers	6.88	3.86	0.89
	Moving light cans	5.80	2.32	0.90
	Moving weight cans	6.03	2.42	0.90
Total alpha dominant hand = 0.91				

**Table 3:** Pearsons' correlation coefficient for nondominant hand between the Jebsen Taylor Hand Function Test (JTHFT) and the Health Assessment Questionnaire (HAQ).

**Correlations non dominant hand**
	**Dressing**	**Arising**	**Eating**	**Walking**	**Hygiene**	**Reaching**	**Grip**	**Activity**
Writing	0.63^**^	0.50^**^	0.33	0.44^*^	0.49^**^	0.37^*^	0.36	0.54^**^
Turning pages	0.69^**^	0.63^**^	0.48^**^	0.57^**^	0.54^**^	0.39^*^	0.20	0.53^**^
Pick up small objects	0.78^**^	0.70^**^	0.44^*^	0.65^**^	0.66^**^	0.54^**^	0.37^*^	0.65^**^
Simulate feeding	0.67^**^	0.56^**^	0.45^*^	0.51^**^	0.57^**^	0.40^*^	0.12	0.60^**^
Stacking Checkers	0.69^**^	0.55^**^	0.47^*^	0.53^**^	0.58^**^	0.38^*^	0.13	0.57^**^
Moving light cans	0.82^**^	0.71^**^	0.57^**^	0.66^**^	0.68^**^	0.53^**^	0.38^*^	0.70^**^
Moving weight cans	0.79^**^	0.68^**^	0.61^**^	0.59^**^	0.68^**^	0.53^**^	0.29	0.68^**^

* *Correlation is significant at the 0.05 level (2-tailed)*.

** *Correlation is significant at the 0.01 level (2-tailed)*.

**Table 4:** Pearsons' correlation coefficient for dominant hand between JTHFT and HAQ.

**Correlations dominant hand**
	**Dressing**	**Arising**	**Eating**	**Walking**	**Hygiene**	**Reaching**	**Grip**	**Activity**
Writing	0.37^*^	0.41^*^	0.32	0.34	0.28	0.31	0.23	0.35
Turning pages	0.77^**^	0.65^**^	0.57^**^	0.58^**^	0.60^**^	0.52^**^	0.59^**^	0.62^**^
Pick up small objects	0.81^**^	0.71^**^	0.58^**^	0.68^**^	0.69^**^	0.54^**^	0.67^**^	0.73^**^
Simulate feeding	0.58^**^	0.49^**^	0.46^*^	0.46^*^	0.46^*^	0.44^*^	0.61^**^	0.46^*^
Stacking checkers	0.75^**^	0.74^**^	0.61^**^	0.70^**^	0.66^**^	0.54^**^	0.27	0.63^**^
Moving light cans	0.84^**^	0.72^**^	0.57^**^	0.63^**^	0.73^**^	0.52^**^	0.40^*^	0.76^**^
Moving weight cans	0.80^**^	0.64^**^	0.64^**^	0.54^**^	0.67^**^	0.47^*^	0.37^*^	0.69^**^

* *Correlation is significant at the 0.05 level (2-tailed)*.

** *Correlation is significant at the 0.01 level (2-tailed)*.

## Discussion

The present study aimed to evaluate the psychometric properties of the JTHFT-IT scale in Italian people with MS. The results of this study have shown that the scale taken into consideration is a reliable and valid tool for the population studied. Cronbach's alpha was used to assess the scale's internal coherence, with values of 0.76 for the non-dominant hand, and 0.91 for the dominant hand. In both cases, the values exceed the minimum value of 0.7 necessary to consider the instrument reliable. Many other studies found the first item (writing) to have an important weight compared to other items, because they considered populations with a specific disorder (Parkinson's disease, stroke, etc.), For this study, however, a difference in time between writing and the other tasks was not found (12, 15, 23).

Interesting results have been found with Pearson's correlation analysis, which reported a statistically significant correlation between the non-dominant and dominant hand items, and the HAQ subtests.

For the non-dominant hand, statistically significant data are given between:

- Item 1 (writing) and the subtest dressing, and arising with a moderate linear relation
- Item 2 and item 3 (page rotation simulation and small object gripping) and all HAQ-scaled subtests with a linear relationship between moderate and strong
- Item 4.5 (simulate feeding and stacking) except for grip 6.7 (collect and lift large, light, and heavy objects) and all HAQ-scaled subtests with a linear relationship between moderate and strong.

As for the dominant hand, on the other hand, they were correlated with a linear relationship between moderate and strong with all JTHFT items except:

- Item 1 (writing) with the subtest “eat” and “grip.”
- Items 2, 4, 5, and 7 (simulate turning a page, simulate feeding, stacking, collecting, and moving large and heavy objects) with “Grip”.

Strong correlations (ρ > 0.70) were found for Jebsen subscales' “pick up small objects,” “moving light cans,” and “moving weight cans” with HAQ subscales' “Dressing,” “Arising,” “Walking,” “Hygiene,” and “Activity”, showing that daily activities are correlated with a better gross motor hand function instead of fine motricity. Another interesting result was that the subscale of “grip” correlates only in the analysis with the dominant hand.

Finally, the correlation between JTHFT and HAQ suggests that a disability related to the upper limbs can often have consequences on walking. This correlation found in literature a study on motor function of the upper and lower extremities and cognitive deterioration in multiple sclerosis (41) showed a statistically significant correlation between a Timed 25 Foot Walk T (T25FW) and an NHPT upper limb function test. The motor deficits that emerged from the T25FW and the NHPT may be linked to a brain marrow disease rather than a spinal disease, even if in the same study (41), we refer to a deepening through MRI that can confirm this hypothesis.

### Limitation of the Study

This study has some limitations. Even though the number of participants is enough to examine the psychometric properties, a larger sample would allow the examination of the influences of the various sociodemographic variables. Finally, the authors agree with previous studies that the JTHFT itself has some limitations (18, 21, 24). The score of the test does not reflect different compensation mechanisms for positioning the upper limb. Hence, it is important to provide appropriate instructions before starting the test and to ask patients to not change their strategy while being tested or, in clinical trials that use the JTHFT score as an endpoint, to not change strategies in follow-up evaluations. Furthermore, the patients with moderate-to-severe functional impairment are often not testable with the JTHFT.

## Conclusion

In conclusion, it can be said that the JTHFT is a reliable tool to evaluate the functionality of the upper limb and hand in patients with MS. The JTHFT, in line with previous studies, is a valid tool for the evaluation of the upper limb. Moreover, in addition to the simplicity and speed of administration, it is an instrument that requires materials that are readily available and can be produced by hand.

However, the appearance of Item 1 must be considered, that is, the writing item, which requires much more time than the other items on the scale. Although no test used in isolation can provide a realistic assessment of hand function, it is important to consider the potential usefulness of JTHFT compared to other tests (12).

## Data Availability Statement

The original contributions presented in the study are included in the article/supplementary material, further inquiries can be directed to the corresponding author.

## Ethics Statement

Ethical review and approval was not required for the study on human participants in accordance with the local legislation and institutional requirements. The patients/participants provided their written informed consent to participate in this study.

## Author Contributions

AB, GG, GF, and AC contributed to conception and design of the study. FP organized the database. GG performed the statistical analysis. AB and FP wrote the first draft of the manuscript. MTo, MTa, VB, and SC wrote sections of the manuscript. All authors contributed to manuscript revision, read, and approved the submitted version.

## Conflict of Interest

The authors declare that the research was conducted in the absence of any commercial or financial relationships that could be construed as a potential conflict of interest.

## Publisher's Note

All claims expressed in this article are solely those of the authors and do not necessarily represent those of their affiliated organizations, or those of the publisher, the editors and the reviewers. Any product that may be evaluated in this article, or claim that may be made by its manufacturer, is not guaranteed or endorsed by the publisher.

## References

[1] Berardelli A Cruccu G. La Neurologia della Sapienza. La Neurologia della Sapienza. (2015) 514–625. 10.15651/978-88-748-8531-2

[2] Lamers I Maris A Severijns D Dielkens W Geurts S Van Wijmeersch B. Upper limb rehabilitation in people with multiple sclerosis: a systematic review. Neurorehabil Neural Repair. (2016) 30:773–93. 10.1177/1545968315624785 26747125

[3] Alonso RN Eizaguirre MB Cohen L Quarracino C Silva B Pita MC. Upper limb dexterity in patients with multiple sclerosis. Int J MS Care. (2021) 23:79–84. 10.7224/1537-2073.2019-083 33880084 PMC8047681

[4] Tramontano M Martino Cinnera A Manzari L Tozzi FF Caltagirone C Morone G. Vestibular rehabilitation has positive effects on balance, fatigue, and activities of daily living in highly disabled multiple sclerosis people: a preliminary randomized controlled trial. Restor Neurol Neurosci. (2018) 36:709–18. 10.3233/RNN-180850 30412513

[5] Tramontano M Morone G De Angelis S Casagrande Conti L Galeoto G Grasso MG. Sensor-based technology for upper limb rehabilitation in patients with multiple sclerosis: a randomized controlled trial. Restor Neurol Neurosci. (2020) 38:333–41. 10.3233/RNN-201033 32925119

[6] Tramontano M Grasso MG Soldi S Casula EP Bonnì S Mastrogiacomo S. Cerebellar intermittent theta-burst stimulation combined with vestibular rehabilitation improves gait and balance in patients with multiple sclerosis: a preliminary double-blind randomized controlled trial. The Cerebellum. (2020) 19:897–901. 10.1007/s12311-020-01166-y 32681455

[7] Kierkegaard M Einarsson U Gottberg K von Koch L Holmqvist LW. The relationship between walking, manual dexterity, cognition and activity/participation in persons with multiple sclerosis. Mult Scler J. (2012) 18:639–46. 10.1177/1352458511426736 21982871

[8] Lamers I Feys P. Assessing upper limb function in multiple sclerosis. Mult Scler J. (2014) 20:775–84. 10.1177/1352458514525677 24664300

[9] Ünlüer NÖ Ozkan T Yaşa ME Ateş Y Anlar Ö. An investigation of upper extremity function in patients with multiple sclerosis, and its relation with shoulder position sense and disability level. Somatosens Mot Res. (2019) 36:189–94. 10.1080/08990220.2019.1644998 31393220

[10] Tofani M Ranieri A Fabbrini G Berardi A Pelosin E Valente D. Efficacy of occupational therapy interventions on quality of life in patients with parkinson's disease: a systematic review and meta-analysis. Mov Disord Clin Pract. (2020) mdc3.13089. 10.1002/mdc3.13089 33163559 PMC7604677

[11] Tofani M Nobilia M Culicchia G Esposito G Savona A Tashi I. The Italian version of rheumatoid arthritis pain scale (IT-RAPS): psychometric properties on community and clinical samples. Reumatismo. (2019) 71:13–8. 10.4081/reumatismo.2019.1043 30932438

[12] Savona A Ferralis L Saffioti M Tofani M Nobilia M Culicchia G. Evaluation of intra- and inter-rater reliability and concurrent validity of the Italian version of the Jebsen–Taylor Hand Function Test in adults with rheumatoid arthritis. Hand Ther. (2019) 24. 10.1177/1758998319843554

[13] Berardi A Saffioti M Tofani M Nobilia M Culicchia G Valente D. Internal consistency and validity of the Jebsen-Taylor hand function test in an Italian population with hemiparesis. Neuro Rehabilitation. (2019) 5:331–9. 10.3233/NRE-192867 31796703

[14] Culicchia G Nobilia M Asturi M Santilli V Paoloni M De Santis R. Cross-cultural adaptation and validation of the jebsen-taylor hand function test in an Italian population. Rehabil Res Pract. (2016) 2016:8970917. 10.1037/t77090-000 27504203 PMC4967698

[15] Ferreiro KN Santos RL. dos, Conforto AB. Psychometric properties of the portuguese version of the Jebsen-Taylor test for adults with mild hemiparesis Brazilian. J Phys Ther. (2010) 14:377–82. 10.1590/S1413-35552010005000018 21203696

[16] Li-Tsang CWP Chan SCC Chan SYY Soo AKW. The Hong Kong Chinese version of the Jebsen Hand Function Test: Inter-rater and test-retest reliabilities. Hong Kong J Occup Ther. (2004) 14:12–20. 10.1016/S1569-1861(09)70024-5

[17] Maas F. An interim australian version of the jebsen test of hand. function. Aust J Physiother. (1982) 28:23–9. 10.1016/S0004-9514(14)60767-4 25025729

[18] Allgöwer K Hermsdörfer J. Fine motor skills predict performance in the Jebsen Taylor Hand Function Test after stroke. Clin Neurophysiol. (2017) 128:1858–71. 10.1016/j.clinph.2017.07.408 28826016

[19] Tofani M Castelli E Sabbadini M Berardi A Murgia M Servadio A. Examining reliability and validity of the jebsen-taylor hand function test among children with cerebral palsy. Percept Mot Skills. (2020) 127:684–97. 10.1177/0031512520920087 32321360

[20] Sigirtmaç IC Öksüz Ç. Investigation of reliability, validity, and cutoff value of the Jebsen-Taylor hand function test. J Hand Ther. (2021) 34:396–403. 10.1016/j.jht.2020.01.004 32156578

[21] Artilheiro MC Fávero FM Caromano FA Oliveira A de SB Carvas N Voos MC. Reliability, validity and description of timed performance of the Jebsen–Taylor Test in patients with muscular dystrophies. Brazilian J Phys Ther. (2018) 22:190–7. 10.1016/j.bjpt.2017.09.010 29292138 PMC5993958

[22] Panuccio F Galeoto G Marquez MA Tofani M Nobilia M Culicchia G. Internal consistency and validity of the Italian version of the Jebsen–Taylor hand function test (JTHFT-IT) in people with tetraplegia. Spinal Cord. (2021) 59:266–73. 10.1038/s41393-020-00602-4 33446935

[23] Mak MKY Lau ETL Tam VWK Woo CWY Yuen SKY. Use of Jebsen Taylor Hand Function Test in evaluating the hand dexterity in people with Parkinson's disease. J Hand Ther. (2015) 28:389–94. 10.1016/j.jht.2015.05.002 26227308

[24] Davis Sears E Chung KC. Validity and responsiveness of the Jebsen-Taylor hand function test. J Hand Surg Am. (2010) 35:30–7. 10.1097/01.prs.0000371796.84278.f7 19954898 PMC4387877

[25] Çelik RGG. Upper extremity capability tests in multiple sclerosis. Noro Psikiyatr Ars. (2018) 55:S54–7. 10.29399/npa.23338 30692857 PMC6278626

[26] Santisteban L Térémetz M Bleton JP Baron JC Maier MA Lindberg PG. Upper limb outcome measures used in stroke rehabilitation studies: a systematic literature review. PLoS ONE. (2016). 10.1371/journal.pone.0154792 27152853 PMC4859525

[27] Jebsen RH Taylor N Trieschmann RB Trotter MJ Howard LA. An objective and standardized test of hand function. Arch Phys Med Rehabil. (1969) 50:311–9. 5788487

[28] Parente M Tofani M De Santis R Esposito G Santilli V Galeoto G. The role of the occupational therapist in disaster areas: systematic review. Occup Ther Int. (2017) 2017:6474761. 10.1155/2017/6474761 29097975 PMC5612682

[29] Berardi A Galeoto G Guarino D Marquez MA De Santis R Valente D. Construct validity, test-retest reliability, and the ability to detect change of the Canadian occupational performance measure in a spinal cord injury population. Spinal cord Ser cases. (2019) 5:52. 10.1038/s41394-019-0196-6 31632710 PMC6786371

[30] Romagnoli G Leone A Sansoni J De Santis R Valente D Galeoto G. Occupational Therapy's efficacy in children with Asperger's syndrome: a systematic review of randomized controlled trials Systematic review. Clin Ter. (2019) 170:382–7. 10.7417/CT.2019.2164 31612197

[31] Murgia M Bernetti A Delicata M Massetti C Achilli EM Mangone M. Inter- and intra-interviewer reliability of Italian version of pediatric evaluation of disability inventory (I-PEDI). Ann Ig. (2018) 30:153–61. 10.7416/ai.2018.2206 29465152

[32] Tofani M Candeloro C Sabbadini M Field D Frascarelli F Lucibello L. A study validating the Italian version of the Level of Sitting Scale in children with cerebral palsy. Clin Rehabil. (2019) 33:1810–8. 10.1177/0269215519858387 31234655

[33] Berardi A Galeoto G Lucibello L Panuccio F Valente D Tofani M. Athletes with disability' satisfaction with sport wheelchairs: an Italian cross sectional study. Disabil Rehabil Assist Technol. (2020) 16:420–4. 10.1080/17483107.2020.1800114 32730722

[34] Fabbri B Berardi A Tofani M Panuccio F Ruotolo I Sellitto G. A systematic review of the psychometric properties of the Jebsen–Taylor hand function test (JTHFT). Hand Surg Rehabil. (2021) 40:560–7. 10.1016/j.hansur.2021.05.004 34023565

[35] McDonald WI Compston A Edan G Goodkin D Hartung HP Lublin FD. Recommended diagnostic criteria for multiple sclerosis: Guidelines from the International Panel on the Diagnosis of Multiple Sclerosis. Ann Neurol. (2001). 10.1002/ana.1032 11456302

[36] Galeoto G De Santis R Marcolini A Cinelli A Cecchi R II. consenso informato in Terapia Occupazionale: Proposta di una modulistica. G Ital Med Lav Ergon. (2016) 38:107–15. 27459843

[37] Galeoto G Mollica R Astorino O Cecchi R. Il consenso informato in fisioterapia: Proposta di una modulistica. G Ital Med Lav Ergon. (2015) 37:245–54. 26934810

[38] Bruce B Fries JF. The health assessment questionnaire (HAQ). Clin Exp Rheumatol. (2005). 23. 16273780

[39] Mokkink LB Terwee CB Patrick DL Alonso J Stratford PW Knol DL. The COSMIN study reached international consensus on taxonomy, terminology, and definitions of measurement properties for health-related patient-reported outcomes. J Clin Epidemiol. (2010) 63:737–45. 10.1016/j.jclinepi.2010.02.006 20494804

[40] Hamilton GF McDonald C Chenier TC. Measurement of grip strength: validity and reliability of the sphygmomanometer and jamar grip dynamometer. J Orthop Sport Phys Ther. (2013) 16:215–9. 10.2519/jospt.1992.16.5.215 18796752

[41] Benedict RHB Holtzer R Motl RW Foley FW Kaur S Hojnacki D. Upper and lower extremity motor function and cognitive impairment in multiple sclerosis. J Int Neuropsychol Soc. (2011) 17:643–53. 10.1017/S1355617711000403 21486517

